# Supplementary material for: Built environment as a risk factor for adult overweight and obesity: Evidence from a longitudinal geospatial analysis in Indonesia
Source: PLOS Glob Public Health. 2022 Oct 5;2(10):e0000481. doi: 10.1371/journal.pgph.0000481 (PMC10021279; doi:10.1371/journal.pgph.0000481)
Supplement: S1 Table — (DOCX) [file pgph.0000481.s001.docx]

| **S1_Table. Linear regression model predicting BMI, Female Sample** (Robust standard errors in parentheses: *** p<0.01, ** p<0.05, * p<0.1) | | | | | | | | |
| --- | --- | --- | --- | --- | --- | --- | --- | --- |
| Variables | Model 1 | Model 2 | Model 3 | Model 4 | Model 5 | Model 6 | Model 7 | Model 8 |
| Percent built-up area of current |  |  | **0.0208***** |  | **0.0208***** |  | **0.0177***** |  |
| residence |  |  | (0.002426) |  | (0.002503) |  | (0.002603) |  |
| Change in % built-up area |  |  |  | 0.0007 |  | 0.0015 |  | 0.0012 |
| since previous panel |  |  |  | (0.002085) |  | (0.002107) |  | (0.002079) |
| Percent built-up area of residence in |  |  |  | **0.0215***** |  | **0.0216***** |  | **0.0185***** |
| previous panel |  |  |  | (0.002533) |  | (0.002626) |  | (0.002730) |
| Current age | **0.1135***** | **0.1124***** | **0.1168***** | **0.1169***** | **0.1136***** | **0.1141***** | **0.1014***** | **0.1017***** |
|  | (0.036081) | (0.036018) | (0.036042) | (0.036063) | (0.035967) | (0.035962) | (0.035590) | (0.035576) |
| Current age squared | **-0.0018***** | **-0.0018***** | **-0.0018***** | **-0.0018***** | **-0.0018***** | **-0.0018***** | **-0.0015***** | **-0.0015***** |
|  | (0.000330) | (0.000330) | (0.000330) | (0.000330) | (0.000329) | (0.000329) | (0.000326) | (0.000326) |
| Island of residence (Ref = Java) |  |  |  |  | *ref* | *ref* | *ref* | *ref* |
| Sumatra |  |  |  |  | 0.2830 | 0.2825 | 0.1798 | 0.1782 |
|  |  |  |  |  | (0.196331) | (0.199920) | (0.198780) | (0.202498) |
| All other islands |  |  |  |  | -0.2123 | -0.2037 | -0.0824 | -0.0729 |
|  |  |  |  |  | (0.182310) | (0.182381) | (0.199236) | (0.199269) |
| Education (Ref = none) |  |  |  |  |  |  | *ref* | *ref* |
| Elementary |  |  |  |  |  |  | **0.9542***** | **0.9580***** |
|  |  |  |  |  |  |  | (0.181021) | (0.180876) |
| Junior high |  |  |  |  |  |  | **1.133***** | **1.140***** |
|  |  |  |  |  |  |  | (0.267148) | (0.266841) |
| Senior high |  |  |  |  |  |  | **1.696***** | **1.703***** |
|  |  |  |  |  |  |  | (0.319508) | (0.319099) |
| College or higher |  |  |  |  |  |  | **1.671***** | **1.676***** |
|  |  |  |  |  |  |  | (0.425598) | (0.425396) |
| Other |  |  |  |  |  |  | **1.202***** | **1.215***** |
|  |  |  |  |  |  |  | (0.367844) | (0.367529) |
| Marital status (Ref = Never married) |  |  |  |  |  |  | *ref* | *ref* |
| Married |  |  |  |  |  |  | 0.8043 | 0.8008 |
|  |  |  |  |  |  |  | (0.722636) | (0.726866) |
| Widowed or other |  |  |  |  |  |  | 0.2815 | 0.2780 |
|  |  |  |  |  |  |  | (0.735320) | (0.739597) |
| Religion (Ref = Islam) |  |  |  |  |  |  | *ref* | *ref* |
| Christianity |  |  |  |  |  |  | -0.2702 | -0.2705 |
|  |  |  |  |  |  |  | (0.309500) | (0.309944) |
| Hindu, Buddhist, or other |  |  |  |  |  |  | 0.0114 | 0.0081 |
|  |  |  |  |  |  |  | (0.333008) | (0.332995) |
| Current smoker (Ref = no) |  |  |  |  |  |  | *ref* | *ref* |
| Yes |  |  |  |  |  |  | -0.1103 | -0.1132 |
|  |  |  |  |  |  |  | (0.290899) | (0.290848) |
| Period (Ref = 1993-2000) | *ref* | *ref* | *ref* | *ref* | *ref* | *ref* | *ref* | *ref* |
| 2000-2007 | **1.371***** | **1.399***** | **1.362***** | **1.373***** | **1.368***** | **1.368***** | **1.275***** | **1.276***** |
|  | (0.078968) | (0.078462) | (0.078704) | (0.080532) | (0.078503) | (0.079904) | (0.080632) | (0.081859) |
| 2007-2014 | **2.449***** | **2.534***** | **2.504***** | **2.515***** | **2.512***** | **2.526***** | **2.308***** | **2.317***** |
|  | (0.124549) | (0.122728) | (0.123149) | (0.123752) | (0.122827) | (0.123463) | (0.129189) | (0.129968) |
| Urban cluster (Ref = rural) | *ref* |  |  |  |  |  |  |  |
| Current urban strata | **1.236***** |  |  |  |  |  |  |  |
|  | (0.136678) |  |  |  |  |  |  |  |
| Previous wave urban strata |  | **1.312***** |  |  |  |  |  |  |
|  |  | (0.140630) |  |  |  |  |  |  |
| Observations (Persons) | 2,306 | 2,306 | 2,306 | 2,306 | 2,306 | 2,306 | 2,306 | 2,306 |
| R^2^ | 0.101 | 0.103 | 0.101 | 0.101 | 0.103 | 0.103 | 0.112 | 0.118 |
