# Supplementary material for: Built environment as a risk factor for adult overweight and obesity: Evidence from a longitudinal geospatial analysis in Indonesia
Source: PLOS Glob Public Health. 2022 Oct 5;2(10):e0000481. doi: 10.1371/journal.pgph.0000481 (PMC10021279; doi:10.1371/journal.pgph.0000481)
Supplement: S2 Table — (DOCX) [file pgph.0000481.s002.docx]

| **S2_Table. Value-added linear regression model predicting BMI, Female Sample** (Robust standard errors in parentheses: *** p<0.01, ** p<0.05, * p<0.1) | | | | | | | | |
| --- | --- | --- | --- | --- | --- | --- | --- | --- |
| Variables | Model 1 | Model 2 | Model 3 | Model 4 | Model 5 | Model 6 | Model 7 | Model 8 |
| Percent built-up area of |  |  | **0.0029***** |  | **0.0026***** |  | **0.0017*** |  |
| current residence |  |  | (0.000905) |  | (0.000940) |  | (0.000970) |  |
| Change in % built-up area |  |  |  | **0.0017*** |  | **0.0019*** |  | 0.0017 |
| since previous panel |  |  |  | (0.001027) |  | (0.001061) |  | (0.001065) |
| Percent built-up area of residence in |  |  |  | **0.0034***** |  | **0.0031***** |  | **0.0022**** |
| previous panel |  |  |  | (0.000950) |  | (0.000999) |  | (0.001032) |
| Current age | **-0.0769***** | **-0.0766***** | **-0.0758***** | **-0.0752***** | **-0.0771***** | **-0.0766***** | **-0.0796***** | **-0.0791***** |
|  | (0.021973) | (0.021985) | (0.021974) | (0.021987) | (0.022011) | (0.022001) | (0.021800) | (0.021796) |
| Current age squared | 0.0002 | 0.0002 | 0.0002 | 0.0002 | 0.0003 | 0.0003 | 0.0003 | 0.0003 |
|  | (0.000210) | (0.000210) | (0.000210) | (0.000210) | (0.000210) | (0.000210) | (0.000207) | (0.000207) |
| Island of residence (Ref = Java) |  |  |  |  | *ref* | *ref* | *ref* | *ref* |
| Sumatra |  |  |  |  | 0.0428 | 0.0752 | 0.0137 | 0.0443 |
|  |  |  |  |  | (0.076196) | (0.078704) | (0.078156) | (0.080781) |
| All other islands |  |  |  |  | **-0.1696**** | **-0.1554**** | -0.1147 | -0.1031 |
|  |  |  |  |  | (0.068460) | (0.068706) | (0.075744) | (0.075840) |
| Education (Ref = none) |  |  |  |  |  |  | *ref* | *ref* |
| Elementary |  |  |  |  |  |  | **0.1852**** | **0.1836**** |
|  |  |  |  |  |  |  | (0.080858) | (0.080939) |
| Junior high |  |  |  |  |  |  | **0.2378**** | **0.2345**** |
|  |  |  |  |  |  |  | (0.114078) | (0.114090) |
| Senior high |  |  |  |  |  |  | **0.5201***** | **0.5158***** |
|  |  |  |  |  |  |  | (0.128920) | (0.128949) |
| College or higher |  |  |  |  |  |  | **0.5463***** | **0.5399***** |
|  |  |  |  |  |  |  | (0.161384) | (0.161562) |
| Other |  |  |  |  |  |  | 0.2644 | 0.2557 |
|  |  |  |  |  |  |  | (0.195345) | (0.195676) |
| Marital status (Ref = Never married) |  |  |  |  |  |  | *ref* | *ref* |
| Married |  |  |  |  |  |  | **0.8647**** | **0.8623**** |
|  |  |  |  |  |  |  | (0.431477) | (0.430606) |
| Widowed or other |  |  |  |  |  |  | 0.7106 | 0.7056 |
|  |  |  |  |  |  |  | (0.435688) | (0.434950) |
| Religion (Ref = Islam) |  |  |  |  |  |  | *ref* | *ref* |
| Christianity |  |  |  |  |  |  | -0.1273 | -0.1181 |
|  |  |  |  |  |  |  | (0.109039) | (0.109361) |
| Hindu, Buddhist, or other |  |  |  |  |  |  | -0.1148 | -0.1079 |
|  |  |  |  |  |  |  | (0.127414) | (0.127859) |
| Current smoker (Ref = no) |  |  |  |  |  |  | *ref* | *ref* |
| Yes |  |  |  |  |  |  | 0.1758 | 0.1711 |
|  |  |  |  |  |  |  | (0.183584) | (0.183920) |
| Period (Ref = 1993-2000) | *ref* | *ref* | *ref* | *ref* | *ref* | *ref* | *ref* | *ref* |
| 2000-2007 | **0.3012***** | **0.3061***** | **0.2998***** | **0.2818***** | **0.3046***** | **0.2841***** | **0.2915***** | **0.2725***** |
|  | (0.070674) | (0.070630) | (0.070774) | (0.071057) | (0.070724) | (0.070885) | (0.070911) | (0.071098) |
| 2007-2014 | **0.3471***** | **0.3629***** | **0.35721***** | **0.3641***** | **0.3656***** | **0.3722***** | **0.3315***** | **0.3382***** |
|  | (0.074646) | (0.074527) | (0.074712) | (0.074700) | (0.074765) | (0.074786) | (0.077356) | (0.077355) |
| Urban cluster (Ref = rural) | ref |  |  |  |  |  |  |  |
| Current urban strata | **0.2167***** |  |  |  |  |  |  |  |
|  | (0.056022) |  |  |  |  |  |  |  |
| Previous wave urban strata |  | **0.2087***** |  |  |  |  |  |  |
|  |  | (0.056120) |  |  |  |  |  |  |
| Lagged BMI | **0.8775***** | **0.8774***** | **0.8783***** | **0.8783***** | **0.8778***** | **0.8778***** | **0.8737***** | **0.8737***** |
|  | (0.012192) | (0.012186) | (0.012109) | (0.012134) | (0.012140) | (0.012157) | (0.012251) | (0.012269) |
| Observations (Persons) | 2,306 | 2,306 | 2,306 | 2,306 | 2,306 | 2,306 | 2,306 | 2,306 |
| R^2^ | 0.660 | 0.659 | 0.659 | 0.659 | 0.660 | 0.660 | 0.661 | 0.661 |
