# Supplementary material for: Built environment as a risk factor for adult overweight and obesity: Evidence from a longitudinal geospatial analysis in Indonesia
Source: PLOS Glob Public Health. 2022 Oct 5;2(10):e0000481. doi: 10.1371/journal.pgph.0000481 (PMC10021279; doi:10.1371/journal.pgph.0000481)
Supplement: S3 Table — (DOCX) [file pgph.0000481.s003.docx]

| **S3_Table. Linear regression model predicting overweight/obese, Female Sample** (Robust standard errors in parentheses: *** p<0.01, ** p<0.05, * p<0.1) | | | | | | | | |
| --- | --- | --- | --- | --- | --- | --- | --- | --- |
| Variables | Model 1 | Model 2 | Model 3 | Model 4 | Model 5 | Model 6 | Model 7 | Model 8 |
| Percent built-up area |  |  | **0.0020***** |  | **0.0020***** |  | **0.0016***** |  |
| of current residence |  |  | (0.000254) |  | (0.000263) |  | (0.000271) |  |
| Change in % built-up area |  |  |  | 0.0001 |  | 0.0002 |  | 0.0002 |
| since previous panel |  |  |  | (0.000241) |  | (0.000242) |  | (0.000233) |
| Percent built-up area of residence |  |  |  | **0.0021***** |  | **0.0021***** |  | **0.0017***** |
| in previous panel |  |  |  | (0.000266) |  | (0.000276) |  | (0.000284) |
| Current age | **0.0137***** | **0.0135***** | **0.0143***** | **0.0143***** | **0.0138***** | **0.0139***** | **0.0123***** | **0.0123***** |
|  | (0.004250) | (0.004242) | (0.004253) | (0.004261) | (0.004236) | (0.004242) | (0.004217) | (0.004222) |
| Current age squared | **-0.0002***** | **-0.0002***** | **-0.0002***** | **-0.0002***** | **-0.0002***** | **-0.0002***** | **-0.0002***** | **-0.0002***** |
|  | (0.000040) | (0.000040) | (0.000040) | (0.000040) | (0.000039) | (0.000040) | (0.000040) | (0.000040) |
| Island of residence (Ref = Java) |  |  |  |  | *ref* | *ref* | *ref* | *ref* |
| Sumatra |  |  |  |  | 0.0356 | **0.0367*** | 0.0228 | 0.0243 |
|  |  |  |  |  | (0.021627) | (0.021845) | (0.021829) | (0.022038) |
| All other islands |  |  |  |  | -0.0313 | -0.0301 | -0.0248 | -0.0235 |
|  |  |  |  |  | (0.021261) | (0.021285) | (0.023037) | (0.023046) |
| Education (Ref = none) |  |  |  |  |  |  | *ref* | *ref* |
| Elementary |  |  |  |  |  |  | **0.1344***** | **0.1347***** |
|  |  |  |  |  |  |  | (0.023175) | (0.023171) |
| Junior high |  |  |  |  |  |  | **0.1735***** | **0.1741***** |
|  |  |  |  |  |  |  | (0.030221) | (0.030189) |
| Senior high |  |  |  |  |  |  | **0.2045***** | **0.2051***** |
|  |  |  |  |  |  |  | (0.033607) | (0.033573) |
| College or higher |  |  |  |  |  |  | **0.2241***** | **0.2243***** |
|  |  |  |  |  |  |  | (0.044732) | (0.044726) |
| Other |  |  |  |  |  |  | **0.1239***** | **0.1245***** |
|  |  |  |  |  |  |  | (0.041271) | (0.041293) |
| Marital status (Ref = Never married) |  |  |  |  |  |  | *ref* | *ref* |
| Married |  |  |  |  |  |  | 0.0455 | 0.0451 |
|  |  |  |  |  |  |  | (0.109038) | (0.109142) |
| Widowed or other |  |  |  |  |  |  | -0.0121 | -0.0126 |
|  |  |  |  |  |  |  | (0.110558) | (0.110670) |
| Religion (Ref = Islam) |  |  |  |  |  |  | *ref* | *ref* |
| Christianity |  |  |  |  |  |  | -0.0328 | -0.0324 |
|  |  |  |  |  |  |  | (0.038483) | (0.038523) |
| Hindu, Buddhist, or other |  |  |  |  |  |  | 0.0435 | 0.0436 |
|  |  |  |  |  |  |  | (0.040064) | (0.040093) |
| Current smoker (yes) |  |  |  |  |  |  | **-0.0766**** | **-0.0771**** |
|  |  |  |  |  |  |  | (0.034768) | (0.034752) |
| Period (Ref = 1993-2000) | ref | ref | ref | ref | ref | ref | ref | ref |
| 2000-2007 | **0.1320***** | **0.1351***** | **0.1315***** | **0.1319***** | **0.1322***** | **0.1314***** | **0.1178***** | **0.1168***** |
|  | (0.010961) | (0.010947) | (0.010966) | (0.011371) | (0.010977) | (0.011376) | (0.011193) | (0.011561) |
| 2007-2014 | **0.2297***** | **0.2388***** | **0.2365***** | **0.2377***** | **0.2376***** | **0.2392***** | **0.2103***** | **0.2116***** |
|  | (0.014671) | (0.014566) | (0.014623) | (0.014636) | (0.014635) | (0.014648) | (0.015339) | (0.015357) |
| Urban cluster (Ref = rural) | ref |  |  |  |  |  |  |  |
| Current urban strata | **0.1340***** |  |  |  |  |  |  |  |
|  | (0.015078) |  |  |  |  |  |  |  |
| Previous wave urban strata |  | **0.1419***** |  |  |  |  |  |  |
|  |  | (0.015383) |  |  |  |  |  |  |
| Observations (Persons) | 2,306 | 2,306 | 2,306 | 2,306 | 2,306 | 2,306 | 2,306 | 2,306 |
| R^2^ | 0.073 | 0.075 | 0.069 | 0.069 | 0.071 | 0.071 | 0.089 | 0.089 |
