# Supplementary material for: Built environment as a risk factor for adult overweight and obesity: Evidence from a longitudinal geospatial analysis in Indonesia
Source: PLOS Glob Public Health. 2022 Oct 5;2(10):e0000481. doi: 10.1371/journal.pgph.0000481 (PMC10021279; doi:10.1371/journal.pgph.0000481)
Supplement: S4 Table — (DOCX) [file pgph.0000481.s004.docx]

| **S4_Table. Value-added linear regression model predicting overweight/obese, Female Sample (**Robust standard errors in parentheses: *** p<0.01, ** p<0.05, * p<0.1) | | | | | | | | |
| --- | --- | --- | --- | --- | --- | --- | --- | --- |
| Variables | Model 1 | Model 2 | Model 3 | Model 4 | Model 5 | Model 6 | Model 7 | Model 8 |
| Percent built-up area of |  |  | **0.0005***** |  | **0.0004**** |  | 0.0002 |  |
| current residence |  |  | (0.000178) |  | (0.000185) |  | (0.000190) |  |
| Change in % built-up area |  |  |  | 0.0002 |  | 0.0002 |  | 0.0002 |
| since previous panel |  |  |  | (0.000181) |  | (0.000184) |  | (0.000181) |
| Percent built-up area of |  |  |  | **0.0005***** |  | **0.0005**** |  | 0.0003 |
| residence in previous panel |  |  |  | (0.000186) |  | (0.000194) |  | (0.000199) |
| Current age | -0.0026 | -0.0026 | -0.0023 | -0.0022 | -0.0025 | -0.0024 | -0.0031 | -0.0030 |
|  | (0.003229) | (0.003231) | (0.003233) | (0.003238) | (0.003225) | (0.003228) | (0.003231) | (0.003234) |
| Current age squared | -0.0000 | -0.0000 | -0.0000 | -0.0000 | -0.0000 | -0.0000 | -0.0000 | -0.0000 |
|  | (0.000030) | (0.000030) | (0.000030) | (0.000030) | (0.000030) | (0.000030) | (0.000030) | (0.000030) |
| Island of residence (Ref = Java) |  |  |  |  | *ref* | *ref* | *ref* | *ref* |
| Sumatra |  |  |  |  | 0.0145 | 0.0189 | 0.0088 | 0.0129 |
|  |  |  |  |  | (0.014390) | (0.014634) | (0.014598) | (0.014816) |
| All other islands |  |  |  |  | **-0.0276*** | **-0.0259*** | **-0.0275*** | **-0.0260*** |
|  |  |  |  |  | (0.014481) | (0.014508) | (0.015758) | (0.015762) |
| Education (Ref = none) |  |  |  |  |  |  | ref | ref |
| Elementary |  |  |  |  |  |  | **0.0692***** | **0.0690***** |
|  |  |  |  |  |  |  | (0.016623) | (0.016637) |
| Junior high |  |  |  |  |  |  | **0.0976***** | **0.0973***** |
|  |  |  |  |  |  |  | (0.021466) | (0.021460) |
| Senior high |  |  |  |  |  |  | **0.1048***** | **0.1044***** |
|  |  |  |  |  |  |  | (0.023859) | (0.023811) |
| College or higher |  |  |  |  |  |  | **0.1286***** | **0.1279***** |
|  |  |  |  |  |  |  | (0.032889) | (0.032850) |
| Other |  |  |  |  |  |  | 0.0443 | 0.0431 |
|  |  |  |  |  |  |  | (0.034903) | (0.035005) |
| Marital status (Ref = Never married) |  |  |  |  |  |  | *ref* | *ref* |
| Married |  |  |  |  |  |  | 0.0506 | 0.0503 |
|  |  |  |  |  |  |  | (0.091941) | (0.091591) |
| Widowed or other |  |  |  |  |  |  | 0.0243 | 0.0236 |
|  |  |  |  |  |  |  | (0.093022) | (0.092681) |
| Religion (Ref = Islam) |  |  |  |  |  |  | *ref* | *ref* |
| Christianity |  |  |  |  |  |  | -0.0207 | -0.0194 |
|  |  |  |  |  |  |  | (0.025641) | (0.025659) |
| Hindu, Buddhist, or other |  |  |  |  |  |  | 0.0328 | 0.0338 |
|  |  |  |  |  |  |  | (0.028762) | (0.028804) |
| Current smoker (Ref = no) |  |  |  |  |  |  | *ref* | *ref* |
| Yes |  |  |  |  |  |  | **-0.0523**** | **-0.0530**** |
|  |  |  |  |  |  |  | (0.024815) | (0.024829) |
| Period (Ref = 1993-2000) | ref | ref | ref | ref | ref | ref | ref | ref |
| 2000-2007 | **0.0435***** | **0.0447***** | **0.0431***** | **0.0410***** | **0.0439***** | **0.0412***** | **0.0378***** | **0.0352***** |
|  | (0.0112) | (0.0112) | (0.0112) | (0.0114) | (0.0112) | (0.0114) | (0.0112) | (0.0115) |
| 2007-2014 | **0.0544***** | **0.0581***** | **0.0565***** | **0.0575***** | **0.0579***** | **0.0589***** | **0.0459***** | **0.0469***** |
|  | (0.0133) | (0.0133) | (0.0133) | (0.0133) | (0.0134) | (0.0134) | (0.0136) | (0.0136) |
| Urban cluster (Ref = rural) | ref |  |  |  |  |  |  |  |
| Current urban strata | **0.0484***** |  |  |  |  |  |  |  |
|  | (0.0107) |  |  |  |  |  |  |  |
| Previous wave urban strata |  | **0.0492***** |  |  |  |  |  |  |
|  |  | (0.0108) |  |  |  |  |  |  |
| Lagged BMI | **0.0750***** | **0.0749***** | **0.0754***** | **0.0754***** | **0.0752***** | **0.0753***** | **0.0741***** | **0.0741***** |
|  | (0.001746) | (0.001749) | (0.001756) | (0.001759) | (0.001757) | (0.001760) | (0.001769) | (0.001772) |
| Observations (Persons) | 2,306 | 2,306 | 2,306 | 2,306 | 2,306 | 2,306 | 2,306 | 2,306 |
| R^2^ | 0.354 | 0.354 | 0.352 | 0.352 | 0.353 | 0.353 | 0.3584 | 0.356 |
