# Supplementary material for: Built environment as a risk factor for adult overweight and obesity: Evidence from a longitudinal geospatial analysis in Indonesia
Source: PLOS Glob Public Health. 2022 Oct 5;2(10):e0000481. doi: 10.1371/journal.pgph.0000481 (PMC10021279; doi:10.1371/journal.pgph.0000481)
Supplement: S5 Table — (DOCX) [file pgph.0000481.s005.docx]

| **S5_Table. Linear regression model predicting BMI, Male Sample** (Robust standard errors in parentheses: *** p<0.01, ** p<0.05, * p<0.1) | | | | | | | | |
| --- | --- | --- | --- | --- | --- | --- | --- | --- |
| Variables | Model 1 | Model 2 | Model 3 | Model 4 | Model 5 | Model 6 | Model 7 | Model 8 |
| Percent built-up area of |  |  | **0.0246***** |  | **0.0263***** |  | **0.0199***** |  |
| current residence |  |  | (0.002686) |  | (0.002809) |  | (0.002805) |  |
| Change in % built-up area |  |  |  | 0.0008 |  | 0.0019 |  | 0.0020 |
| since previous panel |  |  |  | (0.001722) |  | (0.001784) |  | (0.001692) |
| Percent built-up area of residence |  |  |  | **0.0251***** |  | **0.0272***** |  | **0.0207***** |
| in previous panel |  |  |  | (0.002795) |  | (0.002939) |  | (0.002930) |
| Current age | **0.1344***** | **0.1304***** | **0.1352***** | **0.1346***** | **0.1391***** | **0.1389***** | **0.1175***** | **0.1174***** |
|  | (0.034583) | (0.034632) | (0.034734) | (0.034756) | (0.034699) | (0.034727) | (0.033520) | (0.033542) |
| Current age squared | **-0.0015***** | **-0.0015***** | **-0.0015***** | **-0.0015***** | **-0.0015***** | **-0.0015***** | **-0.0013***** | **-0.0013***** |
|  | (0.000299) | (0.000299) | (0.000303) | (0.000303) | (0.000303) | (0.000303) | (0.000292) | (0.000292) |
| Island of residence (Ref = Java) |  |  |  |  | *ref* | *ref* | *ref* | *ref* |
| Sumatra |  |  |  |  | 0.3174 | 0.3184 | 0.1924 | 0.2030 |
|  |  |  |  |  | (0.201925) | (0.206931) | (0.199885) | (0.204225) |
| All other islands |  |  |  |  | **0.4276**** | **0.4341**** | 0.1360 | 0.1461 |
|  |  |  |  |  | (0.187959) | (0.188404) | (0.205955) | (0.206342) |
| Education (Ref = none) |  |  |  |  |  |  | ref | ref |
| Elementary |  |  |  |  |  |  | **0.7979***** | **0.8059***** |
|  |  |  |  |  |  |  | (0.185820) | (0.185631) |
| Junior high |  |  |  |  |  |  | **1.431***** | **1.446***** |
|  |  |  |  |  |  |  | (0.280058) | (0.279595) |
| Senior high |  |  |  |  |  |  | **2.107***** | **2.123***** |
|  |  |  |  |  |  |  | (0.256709) | (0.256261) |
| College or higher |  |  |  |  |  |  | **2.469***** | **2.478***** |
|  |  |  |  |  |  |  | (0.337042) | (0.337302) |
| Other |  |  |  |  |  |  | **1.219***** | **1.227***** |
|  |  |  |  |  |  |  | (0.380047) | (0.379628) |
| Marital status (Ref = Never married) |  |  |  |  |  |  | *ref* | *ref* |
| Married |  |  |  |  |  |  | **1.268*** | **1.270*** |
|  |  |  |  |  |  |  | (0.691719) | (0.696259) |
| Widowed or other |  |  |  |  |  |  | 0.9112 | 0.9114 |
|  |  |  |  |  |  |  | (0.718152) | (0.722628) |
| Religion (Ref = Islam) |  |  |  |  |  |  | *ref* | *ref* |
| Christianity |  |  |  |  |  |  | -0.0398 | -0.0423 |
|  |  |  |  |  |  |  | (0.292476) | (0.292250) |
| Hindu, Buddhist, or other |  |  |  |  |  |  | 0.4088 | 0.4081 |
|  |  |  |  |  |  |  | (0.324154) | (0.324111) |
| Current smoker (Ref = no) |  |  |  |  |  |  | *ref* | *ref* |
| Yes |  |  |  |  |  |  | **-0.8961***** | **-0.8977***** |
|  |  |  |  |  |  |  | (0.143400) | (0.143475) |
| Period (Ref = 1993-2000) | *ref* | *ref* | *ref* | *ref* | *ref* | *ref* | *ref* | *ref* |
| 2000-2007 | **0.8286***** | **0.8834***** | **0.8201***** | **0.8338***** | **0.8051***** | **0.8063***** | **0.7395***** | **0.7340***** |
|  | (0.077759) | (0.077734) | (0.077637) | (0.082082) | (0.077654) | (0.082623) | (0.076230) | (0.080865) |
| 2007-2014 | **1.251***** | **1.350***** | **1.301***** | **1.317***** | **1.274***** | **1.295***** | **1.274***** | **1.291***** |
|  | (0.122045) | (0.121774) | (0.121764) | (0.121296) | (0.121915) | (0.121300) | (0.121605) | (0.121047) |
| Urban cluster (Ref = rural) | ref |  |  |  |  |  |  |  |
| Current urban strata | **1.281***** |  |  |  |  |  |  |  |
|  | (0.141966) |  |  |  |  |  |  |  |
| Previous wave urban strata |  | **1.389***** |  |  |  |  |  |  |
|  |  | (0.149108) |  |  |  |  |  |  |
| Observations (Persons) | 1,464 | 1,464 | 1,464 | 1,464 | 1,464 | 1,464 | 1,464 | 1,464 |
| R^2^ | 0.079 | 0.084 | 0.090 | 0.090 | 0.094 | 0.093 | 0.159 | 0.159 |
