# Supplementary material for: Built environment as a risk factor for adult overweight and obesity: Evidence from a longitudinal geospatial analysis in Indonesia
Source: PLOS Glob Public Health. 2022 Oct 5;2(10):e0000481. doi: 10.1371/journal.pgph.0000481 (PMC10021279; doi:10.1371/journal.pgph.0000481)
Supplement: S6 Table — (DOCX) [file pgph.0000481.s006.docx]

| **S6_Table. Value-added linear regression model predicting BMI, Male Sample** (Robust standard errors in parentheses: *** p<0.01, ** p<0.05, * p<0.1) | | | | | | | | |
| --- | --- | --- | --- | --- | --- | --- | --- | --- |
| Variables | Model 1 | Model 2 | Model 3 | Model 4 | Model 5 | Model 6 | Model 7 | Model 8 |
| Percent built-up area |  |  | **0.0065***** |  | **0.0068***** |  | **0.0053***** |  |
| of current residence |  |  | (0.001059) |  | (0.001104) |  | (0.001102) |  |
| Change in % built-up area |  |  |  | 0.0010 |  | **0.0015*** |  | **0.0015*** |
| since previous panel |  |  |  | (0.000789) |  | (0.000818) |  | (0.000798) |
| Percent built-up area of |  |  |  | **0.0068***** |  | **0.0073***** |  | **0.0057***** |
| residence in the previous panel |  |  |  | (0.001100) |  | (0.001153) |  | (0.001153) |
| Current age | -0.0144 | -0.0147 | -0.0132 | -0.0132 | -0.0138 | -0.0138 | -0.0146 | -0.0145 |
|  | (0.018696) | (0.018632) | (0.018615) | (0.018626) | (0.018656) | (0.018668) | (0.018651) | (0.018666) |
| Current age squared | -0.0001 | -0.0001 | -0.0001 | -0.0001 | -0.0001 | -0.0001 | -0.0001 | -0.0001 |
|  | (0.000167) | (0.000166) | (0.000166) | (0.000167) | (0.000167) | (0.000167) | (0.000167) | (0.000167) |
| Island of residence (Ref = Java) |  |  |  |  | *ref* | *ref* | *ref* | *ref* |
| Sumatra |  |  |  |  | **0.1345*** | **0.1544**** | **0.1004** | **0.1217*** |
|  |  |  |  |  | (0.070069) | (0.072358) | (0.071401) | (0.073441) |
| All other islands |  |  |  |  | 0.0348 | 0.0437 | -0.0048 | 0.0037 |
|  |  |  |  |  | (0.066202) | (0.066449) | (0.072806) | (0.072891) |
| Education (Ref = none) |  |  |  |  |  |  | ref | ref |
| Elementary |  |  |  |  |  |  | **0.4093***** | **0.4102***** |
|  |  |  |  |  |  |  | (0.081956) | (0.082058) |
| Junior high |  |  |  |  |  |  | **0.5624***** | **0.5649***** |
|  |  |  |  |  |  |  | (0.117208) | (0.117049) |
| Senior high |  |  |  |  |  |  | **0.8006***** | **0.8041***** |
|  |  |  |  |  |  |  | (0.114227) | (0.114296) |
| College or higher |  |  |  |  |  |  | **0.7035***** | **0.7041***** |
|  |  |  |  |  |  |  | (0.126317) | (0.126292) |
| Other |  |  |  |  |  |  | **0.4732**** | **0.4712**** |
|  |  |  |  |  |  |  | (0.216986) | (0.217593) |
| Marital status (Ref = Never married) |  |  |  |  |  |  | *ref* | *ref* |
| Married |  |  |  |  |  |  | 0.1730 | 0.1745 |
|  |  |  |  |  |  |  | (0.245844) | (0.247144) |
| Widowed or other |  |  |  |  |  |  | 0.115 | 0.1136 |
|  |  |  |  |  |  |  | (0.267141) | (0.268576) |
| Religion (Ref = Islam) |  |  |  |  |  |  | *ref* | *ref* |
| Christianity |  |  |  |  |  |  | 0.0219 | 0.0269 |
|  |  |  |  |  |  |  | (0.111787) | (0.112007) |
| Hindu, Buddhist, or other |  |  |  |  |  |  | 0.0362 | 0.0402 |
|  |  |  |  |  |  |  | (0.120177) | (0.120177) |
| Current smoker (Ref = no) |  |  |  |  |  |  | *ref* | *ref* |
| Yes |  |  |  |  |  |  | **-0.3599***** | **-0.3595***** |
|  |  |  |  |  |  |  | (0.062205) | (0.062240) |
| Period (Ref = 1993-2000) | *ref* | *ref* | *ref* | *ref* | *ref* | *ref* | *ref* | *ref* |
| 2000-2007 | **0.4700***** | **0.4856***** | **0.4697***** | **0.4633***** | **0.4686***** | **0.4566***** | **0.4494***** | **0.4364***** |
|  | (0.069191) | (0.069460) | (0.069199) | (0.070451) | (0.069307) | (0.070567) | (0.068491) | (0.069604) |
| 2007-2014 | **0.1977***** | **0.2285***** | **0.2176***** | **0.2244***** | **0.2150***** | **0.2230***** | **0.2398***** | **0.2473***** |
|  | (0.065944) | (0.065787) | (0.065865) | (0.065820) | (0.065953) | (0.065877) | (0.067422) | (0.067368) |
| Urban cluster (Ref = rural) | *ref* |  |  |  |  |  |  |  |
| Current urban strata | **0.370***** |  |  |  |  |  |  |  |
|  | (0.0568) |  |  |  |  |  |  |  |
| Previous wave urban strata |  | **0.363***** |  |  |  |  |  |  |
|  |  | (0.0592) |  |  |  |  |  |  |
| Lagged BMI | **0.9066***** | **0.9057***** | **0.9039***** | **0.9041***** | **0.9034***** | **0.9036***** | **0.8822***** | **0.8822***** |
|  | (0.020185) | (0.020292) | (0.020427) | (0.020409) | (0.020533) | (0.020509) | (0.021305) | (0.021285) |
| Observations (Persons) | 1,464 | 1,464 | 1,464 | 1,464 | 1,464 | 1,464 | 1,464 | 1,464 |
| R^2^ | 0.677 | 0.677 | 0.678 | 0.678 | 0.678 | 0.678 | 0.684 | 0.685 |
