# Supplementary material for: Built environment as a risk factor for adult overweight and obesity: Evidence from a longitudinal geospatial analysis in Indonesia
Source: PLOS Glob Public Health. 2022 Oct 5;2(10):e0000481. doi: 10.1371/journal.pgph.0000481 (PMC10021279; doi:10.1371/journal.pgph.0000481)
Supplement: S7 Table — (DOCX) [file pgph.0000481.s007.docx]

| **S7_Table. Linear regression model predicting overweight/obese, Male Sample** (Robust standard errors in parentheses: *** p<0.01, ** p<0.05, * p<0.1) | | | | | | | | |
| --- | --- | --- | --- | --- | --- | --- | --- | --- |
| Variables | Model 1 | Model 2 | Model 3 | Model 4 | Model 5 | Model 6 | Model 7 | Model 8 |
| Percent built-up area of |  |  | **0.0033***** |  | **0.0036***** |  | **0.0027***** |  |
| current residence |  |  | (0.000365) |  | (0.000380) |  | (0.000384) |  |
| Change in % built-up area since |  |  |  | 0.0002 |  | 0.0003 |  | 0.0003 |
| previous panel |  |  |  | (0.000283) |  | (0.000287) |  | (0.000280) |
| Percent built up area of residence in |  |  |  | **0.0034***** |  | **0.0037***** |  | **0.0028***** |
| previous panel |  |  |  | (0.000380) |  | (0.000398) |  | (0.000401) |
| Current age | **0.0194***** | **0.0189***** | **0.0196***** | **0.0196***** | **0.0203***** | **0.0203***** | **0.0175***** | **0.0176***** |
|  | (0.005512) | (0.005527) | (0.005575) | (0.005579) | (0.005598) | (0.005601) | (0.005463) | (0.005467) |
| Current age squared | **-0.0002***** | **-0.0002***** | **-0.0002***** | **-0.0002***** | **-0.0002***** | **-0.0002***** | **-0.0002***** | **-0.0002***** |
|  | (0.000049) | (0.000049) | (0.000050) | (0.000050) | (0.000050) | (0.000050) | (0.000049) | (0.000049) |
| Island of residence (Ref = Java) |  |  |  |  | *ref* | *ref* | *ref* | *ref* |
| Sumatra |  |  |  |  | **0.0529*** | **0.0542*** | 0.0362 | 0.0387 |
|  |  |  |  |  | (0.030566) | (0.031065) | (0.030369) | (0.030795) |
| All other islands |  |  |  |  | **0.0700***** | **0.0708***** | 0.0296 | 0.0307 |
|  |  |  |  |  | (0.026926) | (0.026996) | (0.029618) | (0.029649) |
| Education (Ref = none) |  |  |  |  |  |  | *ref* | *ref* |
| Elementary |  |  |  |  |  |  | **0.1341***** | **0.1352***** |
|  |  |  |  |  |  |  | (0.029652) | (0.029634) |
| Junior high |  |  |  |  |  |  | **0.1775***** | **0.1800***** |
|  |  |  |  |  |  |  | (0.040318) | (0.040227) |
| Senior high |  |  |  |  |  |  | **0.3097***** | **0.3125***** |
|  |  |  |  |  |  |  | (0.039484) | (0.039430) |
| College or higher |  |  |  |  |  |  | **0.3895***** | **0.3912***** |
|  |  |  |  |  |  |  | (0.048422) | (0.048422) |
| Other |  |  |  |  |  |  | **0.1425**** | **0.1433**** |
|  |  |  |  |  |  |  | (0.058473) | (0.058497) |
| Marital status (Ref = Never married) |  |  |  |  |  |  | *ref* | *ref* |
| Married |  |  |  |  |  |  | 0.1019 | 0.1017 |
|  |  |  |  |  |  |  | (0.113782) | (0.114454) |
| Widowed or other |  |  |  |  |  |  | 0.0588 | 0.0581 |
|  |  |  |  |  |  |  | (0.119256) | (0.119908) |
| Religion (Ref = Islam) |  |  |  |  |  |  | *ref* | *ref* |
| Christianity |  |  |  |  |  |  | 0.0036 | 0.0034 |
|  |  |  |  |  |  |  | (0.044780) | (0.044774) |
| Hindu, Buddhist, or other |  |  |  |  |  |  | 0.0508 | 0.0512 |
|  |  |  |  |  |  |  | (0.045991) | (0.046053) |
| Current smoker (Ref = no) |  |  |  |  |  |  | *ref* | *ref* |
| Yes |  |  |  |  |  |  | **-0.1410***** | **-0.1413***** |
|  |  |  |  |  |  |  | (0.021603) | (0.021608) |
| Period (Ref = 1993-2000) | *ref* | *ref* | *ref* | *ref* | *ref* | *ref* | *ref* | *ref* |
| 2000-2007 | **0.1188***** | **0.1263***** | **0.1178***** | **0.1191***** | **0.1153***** | **0.1145***** | **0.1051***** | **0.1034***** |
|  | (0.013934) | (0.013868) | (0.013915) | (0.014332) | (0.013939) | (0.014386) | (0.013777) | (0.014191) |
| 2007-2014 | **0.1758***** | **0.1898***** | **0.1831***** | **0.1856***** | **0.1788***** | **0.1820***** | **0.1813***** | **0.1840***** |
|  | (0.020279) | (0.020068) | (0.020074) | (0.020057) | (0.020122) | (0.020093) | (0.020021) | (0.020012) |
| Urban cluster (Ref = rural) | *ref* |  |  |  |  |  |  |  |
| Current urban strata | **0.1776***** |  |  |  |  |  |  |  |
|  | (0.020878) |  |  |  |  |  |  |  |
| Previous wave urban strata |  | **0.1892***** |  |  |  |  |  |  |
|  |  | (0.021668) |  |  |  |  |  |  |
| Observations (Persons) | 1,464 | 1,464 | 1,464 | 1,464 | 1,464 | 1,464 | 1,464 | 1,464 |
| R^2^ | 0.062 | 0.066 | 0.069 | 0.068 | 0.073 | 0.072 | 0.131 | 0.130 |
