# Supplementary material for: Built environment as a risk factor for adult overweight and obesity: Evidence from a longitudinal geospatial analysis in Indonesia
Source: PLOS Glob Public Health. 2022 Oct 5;2(10):e0000481. doi: 10.1371/journal.pgph.0000481 (PMC10021279; doi:10.1371/journal.pgph.0000481)
Supplement: S8 Table — (DOCX) [file pgph.0000481.s008.docx]

| **S8_Table. Value-added linear regression model predicting overweight/obese, Male Sample** (Robust standard errors in parentheses: *** p<0.01, ** p<0.05, * p<0.1) | | | | | | | | |
| --- | --- | --- | --- | --- | --- | --- | --- | --- |
| Variables | Model 1 | Model 2 | Model 3 | Model 4 | Model 5 | Model 6 | Model 7 | Model 8 |
| Percent built-up area of |  |  | **0.0010***** |  | **0.0011***** |  | **0.0009***** |  |
| current residence |  |  | (0.000231) |  | (0.000240) |  | (0.000242) |  |
| Change in % built-up area |  |  |  | 0.0002 |  | 0.0003 |  | 0.0003 |
| since previous panel |  |  |  | (0.000211) |  | (0.000215) |  | (0.000213) |
| Percent built-up area of residence |  |  |  | **0.0010***** |  | **0.0012***** |  | **0.009***** |
| in previous panel |  |  |  | (0.000239) |  | (0.000251) |  | (0.000252) |
| Current age | 0.0007 | 0.0006 | 0.0009 | 0.0009 | 0.0010 | 0.0010 | 0.0011 | 0.0012 |
|  | (0.004005) | (0.004006) | (0.004024) | (0.004027) | (0.004043) | (0.004046) | (0.004013) | (0.004017) |
| Current age squared | -0.0000 | -0.0000 | -0.0000 | -0.0000 | -0.0000 | -0.0000 | -0.0000 | -0.0000 |
|  | (0.000036) | (0.000036) | (0.000036) | (0.000036) | (0.000036) | (0.000036) | (0.000036) | (0.000036) |
| Island of residence (Ref = Java) |  |  |  |  | *ref* | *ref* | *ref* | *ref* |
| Sumatra |  |  |  |  | 0.0299 | **0.0335*** | 0.0248 | 0.0286 |
|  |  |  |  |  | (0.019361) | (0.019771) | (0.019650) | (0.020033) |
| All other islands |  |  |  |  | 0.0204 | 0.0216 | 0.0121 | 0.0130 |
|  |  |  |  |  | (0.015518) | (0.015567) | (0.017469) | (0.017486) |
| Education (Ref = none) |  |  |  |  |  |  | *ref* | *ref* |
| Elementary |  |  |  |  |  |  | **0.0857***** | **0.0859***** |
|  |  |  |  |  |  |  | (0.019912) | (0.019926) |
| Junior high |  |  |  |  |  |  | **0.0694***** | **0.0703***** |
|  |  |  |  |  |  |  | (0.026404) | (0.026360) |
| Senior high |  |  |  |  |  |  | **0.1472***** | **0.1483***** |
|  |  |  |  |  |  |  | (0.026919) | (0.026921) |
| College or higher |  |  |  |  |  |  | **0.1698***** | **0.1703***** |
|  |  |  |  |  |  |  | (0.032379) | (0.032344) |
| Other |  |  |  |  |  |  | 0.0496 | 0.0491 |
|  |  |  |  |  |  |  | (0.045395) | (0.045565) |
| Marital status (Ref = Never married) |  |  |  |  |  |  | *ref* | *ref* |
| Married |  |  |  |  |  |  | -0.0344 | -0.0347 |
|  |  |  |  |  |  |  | (0.070937) | (0.071295) |
| Widowed or other |  |  |  |  |  |  | -0.0401 | -0.0412 |
|  |  |  |  |  |  |  | (0.075682) | (0.076052) |
| Religion (Ref = Islam) |  |  |  |  |  |  | *ref* | *ref* |
| Christianity |  |  |  |  |  |  | 0.0113 | 0.0121 |
|  |  |  |  |  |  |  | (0.027965) | (0.027943) |
| Hindu, Buddhist, or other |  |  |  |  |  |  | 0.0044 | 0.0054 |
|  |  |  |  |  |  |  | (0.026870) | (0.026888) |
| Current smoker (Ref = no) |  |  |  |  |  |  | *ref* | *ref* |
| Yes |  |  |  |  |  |  | **-0.0742***** | **-0.0743***** |
|  |  |  |  |  |  |  | (0.014180) | (0.014184) |
| Period (Ref = 1993-2000) | *ref* | *ref* | *ref* | *ref* | *ref* | *ref* | *ref* | *ref* |
| 2000-2007 | **0.0735***** | **0.0761***** | **0.0735***** | **0.0723***** | **0.0729***** | **0.0704***** | **0.0691***** | **0.0663***** |
|  | (0.013084) | (0.013086) | (0.013085) | (0.013264) | (0.013101) | (0.013297) | (0.012968) | (0.013141) |
| 2007-2014 | **0.0430***** | **0.0482***** | **0.0464***** | **0.0475***** | **0.0453***** | **0.0468***** | **0.0529***** | **0.0540***** |
|  | (0.015677) | (0.015651) | (0.015638) | (0.015676) | (0.015658) | (0.015686) | (0.015864) | (0.015904) |
| Urban cluster (Ref = rural) | *ref* |  |  |  |  |  |  |  |
| Current urban strata | **0.0623***** |  |  |  |  |  |  |  |
|  | (0.013593) |  |  |  |  |  |  |  |
| Previous wave urban strata |  | **0.0596***** |  |  |  |  |  |  |
|  |  | (0.013893) |  |  |  |  |  |  |
| Lagged BMI | **0.1144***** | **0.1143***** | **0.1141***** | **0.1142***** | **0.1139***** | **0.1140***** | **0.1098***** | **0.1098***** |
|  | (0.003133) | (0.003154) | (0.003162) | (0.003163) | (0.003176) | (0.003179) | (0.003276) | (0.003280) |
| Observations (Persons) | 1,464 | 1,464 | 1,464 | 1,464 | 1,464 | 1,464 | 1,464 | 1,464 |
| R^2^ | 0.450 | 0.450 | 0.450 | 0.450 | 0.450 | 0.450 | 0.462 | 0.462 |
