# Supplementary material for: Built environment as a risk factor for adult overweight and obesity: Evidence from a longitudinal geospatial analysis in Indonesia
Source: PLOS Glob Public Health. 2022 Oct 5;2(10):e0000481. doi: 10.1371/journal.pgph.0000481 (PMC10021279; doi:10.1371/journal.pgph.0000481)
Supplement: Attachment — Submitted filename: Response to reviewers.pdf [file pgph.0000481.s010.pdf]

Responses to Reviewer 1 comments:

**Reviewer #1 comments**	**Response to Reviewer #1 comments**
1.The authors argued that the longitudinal data are a major contribution of this study. However, they do not consider fixed effects model or other models for panel data. Thus, their results do not have new contributions compared to the cross-sectional design. They argued that fixed effects models are very inefficient. However, the fixed effect models can remove the effects of time-invariant variables, providing more accurate estimations.	For the reasons stated in our last response, we respectfully disagree with the reviewer on the use of FE for this approach. The statistical analyses conducted here include value-added models that efficiently use the panel nature of these data (i.e. they utilize the cross-sectional and longitudinal variation that exists in the estimation of the regression coefficients as well as the clustered standard errors).
2. I believe developing countries (e.g. Indonesia) are important. In particular, the authors argued that "part of the contribution of this paper is to compare how commonly used indicators of urbanization (based on simple urban-rural dichotomies) perform in comparison to more sophisticated measures of urbanization (based on built-up)." However, the authors need to highlight the NEW findings from urbanization (based on built-up)." However, the authors need to highlight the NEW findings from Indonesia and explain why these findings are different from other contexts. At least, I do not see any NEW finding in this version.	Please see our conclusion where the implications of urbanization based on built-up are further drawn on, based on recent studies.
3. The 2km (or 5km, 10km) buffers are too large because people's walking distances are about 1km. Most previous studies use 1/4 or 1/2 miles buffers.	The buffers are not intended to measure a walkability score, but rather the built-up character (proxying for urban) of neighborhoods. We have added some text and a reference by way of explanation.

Responses to Reviewer 3 comments:

**Reviewer #3 comments**	**Response to Reviewer #3 comments**
Page 2, line 9: "gross domestic product (GDP), has doubled from $4.8K to $11.1K". Please specify the currency and what "K" stands for.	We have edited this to reflect 1000s and currency; specifically, USD $4,800 and USD $11,100.
Page 6, Line 123: "Two trained nurses assessed all individuals for health measurements during the survey, unless participants were too ill or pregnant". What classify as "too ill"? Are pregnant women excluded from analysis? How is pregnancy-related (including post pregnancy) overweight and obesity controlled in this study?	We have slightly edited this text. The survey documents do not specify what was considered 'too ill'. We imagine that might have been left to the discretion of the nurse. We excluded pregnant women from the analysis but not post-partum. (We do not control for post-partum status; higher average weights post pregnancy would be controlled for as part of overall sex differences (variable 'woman' in Tables 3-4).)
Inconsistent terminology. Throughout the document the terms gender/sex, men/male and women/female are used interchangeably (such as Page 43 line 458 "Gender-stratified tables"). However, I believe the data set uses biological sex, not gender.	This is a good point and we have made some changes to be consistent. Gender has been replaced by sex and male/female by men/women.
Table 3a - 4b are confusing. instead of Models 1 - 8, are you able to change the headers that reflect the actual models?	We have updated these to reflect model content.
The many tables on sociodemographic factors are fascinating not that necessary. Since this paper focuses on built environment, can you omit some of the information that's not too relevant?	Sociodemographics appear as part of Table 2 (sample descriptives) and Tables 3-4 (main results). We believe showing this level of detail is useful to understand sample composition and the extent to which individual-level factors explain how urbanization affects weight. However, we note that Tables 4a and 4b are quite similar to Tables 3a and 3b in terms of the role of the sociodemographic covariates. If space is a concern, we could omit those coefficients and show full results in an appendix only, which would shorten Tables 4a and 4b. We will consult with the journal's editorial team.

Responses to Reviewer 4 comments:

Reviewer #4 comments	Response to Reviewer #4 comments
My major comment is on a few variables that are considered important for this relationship but were missing in your discussion. In the discussion section on line 497, you mention that your understanding of the drivers of overweight and obesity over time in the context of unplanned and rapid urbanization is still evolving, however, I urge you to review the literature on how neighborhood crime, walkability scores, bike-ability, perceptions of neighborhood safety, access to public transportation, density of amenities and street lighting, and how these potentially affect physical activity.	Noted – we have updated the discussion to point to recent but limited evidence placing Indonesia and other Asian cities in a global context. We note the lack of studies in low and middle-income countries, and the need for them, particularly as connected to health outcomes.
I am also curious on how your analysis considered participant migration in and out of their primary areas of residence. Is it possible that for some participants, they did not live fully in their primary locations as reported by the data? I may have missed this in your discussion of the data, but if otherwise, then this is a factor that could greatly skew the results.	While it is true that people migrate for work, we were relying on measurements taken at 'home', i.e. the address registered for each person over several panels. With the exception of perhaps a very small number of individuals who might have been interviewed during a home visit from a work location in another area, we did not think that migration or moving was a significant issue for this cohort as we were looking at repeat measurements at the same address over several years. This has been noted in the methods section.

Responses to Reviewer 5 comments

**Reviewer #5 comments**	**Response to Reviewer #5 comments**
The background Objective, methods and findings were adequate. However, there is need to recast the last paragraph and include what the result/what was found mean. What are the authors implying with what has been seen in the study? What is the implication on the health of the urban population?	We have edited the final paragraph to reflect the role of cultural difference and access based on social identity in how urban people use public spaces – especially for physical activity.
