# Supplementary material for: Built environment as a risk factor for adult overweight and obesity: Evidence from a longitudinal geospatial analysis in Indonesia
Source: PLOS Glob Public Health. 2022 Oct 5;2(10):e0000481. doi: 10.1371/journal.pgph.0000481 (PMC10021279; doi:10.1371/journal.pgph.0000481)
Supplement: Attachment — Submitted filename: Response to reviewer 2 comments.docx [file pgph.0000481.s009.docx]

**Response to reviewer 2 comments:**

| Reviewer # 2 comment | Authors’ response (line numbers refer to the unmarked Manuscript version) |
| --- | --- |
| 1. It is unclear why a 2 km radius of the respondent's home was used to measure the built-up area. | We examined several different radii when conducting data analysis (2, 5, and 10) and found the results to be not qualitatively different. We chose 2 km because we felt conditions most proximal to a respondents' home would have the greatest impact on obesity risk. |
| 2. More importantly, the authors only used fixed-effect models. Given the nature of panel data, mixed models could be applied here. However, it is unclear why the authors did not use them. | The manuscript does not use fixed-effect models. Instead, linear regression models were fitted that accounted for the longitudinal nature of the data when calculating standard errors. |
